# Vegan Sesame Milk Ice Cream With Probiotic Bacteria (*Lactobacillus casei*) and Hibiscus (*Hibiscus sabdariffa*) Calyces Extract: Formulation, Characterization, and Stability

**DOI:** 10.1002/fsn3.71256

**Published:** 2025-12-25

**Authors:** Zohreh Didar, Mohammad Hossein Haddad Khodaparast, Behzad Goharjoo

**Affiliations:** ^1^ Department of Food Science and Technology Ne.C., Islamic Azad University Neyshabur Iran; ^2^ Department of Food Science and Technology, Faculty of Agriculture Ferdowsi University of Mashhad Mashhad Iran; ^3^ Bachelor Student in TEFL Farhangian University Mashhad Iran

**Keywords:** *Hibiscus sabdariffa*, probiotic, vegan ice cream

## Abstract

In this study, probiotic ice cream was created using sesame seed milk and enriched with co‐extruded microcapsules containing probiotic bacteria ( *Lactobacillus casei* ) and Hibiscus ( *Hibiscus sabdariffa* ) calyces extract. Results showed no significant difference in fat content between the control and enriched samples (both about 2.1%, *p* > 0.05). However, enrichment significantly reduced hardness (98 ± 2 N vs. 90 ± 2 N) and increased overrun (22.42% ± 1.2% vs. 28.42% ± 1.5%) and melting resistance (80.2% ± 0.5% vs. 88.2% ± 0.2%) (*p* < 0.05). The enriched ice cream contained higher anthocyanin (0.25 ± 0.01 mg/100 g vs. none detected in control), total flavonol (7.57 ± 0.1 mg/100 g vs. 0.7 ± 0.01 mg/100 g), ascorbic acid (0.14 ± 0.01 mg/100 g vs. 0.06 ± 0.01 mg/100 g), total phenolic content (3.72 ± 0.3 mg GAE/100 g vs. 0.25 ± 0.02 mg GAE/100 g), and antioxidant activity (31% ± 1.2% vs. 10.5% ± 2.5%) (*p* < 0.05). The viability of probiotic bacteria decreased over the 45‐day storage period, reaching a logarithmic viable cell count of 6 ± 0.01 CFU/g at the end. While TPC content remained stable, anthocyanin, total flavonol, and ascorbic acid content showed significant decreases by the end of the storage (*p* < 0.05). In simulated gastrointestinal conditions, the logarithmic number of co‐extruded probiotic bacterial cells decreased from 8 to 7.1 CFU/g after 1 h, and in simulated intestinal juice, the logarithmic viable cells reached 6.2 CFU/g. Sensory evaluation revealed a significant difference in appearance, flavor, and general acceptability between the control and enriched samples (*p* < 0.05), with the enriched samples scoring higher in these categories. In conclusion, this study demonstrates that co‐extrusion technology effectively improves bioactive compound retention, probiotic stability, and sensory quality in plant‐based probiotic ice cream.

## Graphical abstract

Formulation and characterization vegan ice cream based on sesame milk included co‐extruded probiotic bacteria (*Lactobacillus casei*) and Hibiscus (*Hibiscus sabdariffa*) Calyces extract, stability during storage and simulated gastrointestinal conditions.

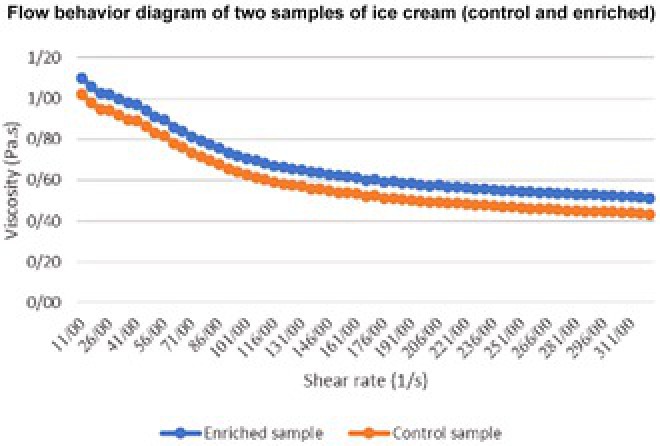

## 1 Introduction

Plant‐based milk is increasingly consumed by individuals with lactose intolerance, cow's milk allergy such as β‐lg (Gao et al. 2022), or dietary preferences such as vegetarianism (Cui et al. 2023). Cereals, legumes, seeds, nuts, and pseudocereals have been widely used to prepare such milk alternatives, and their application in diverse food products has become a growing research focus, especially in frozen desserts like ice cream. Ice cream is a complex frozen matrix containing proteins, vitamins, minerals, and bioactive compounds. Plant‐based versions have been successfully developed from hazelnut, walnut, soy, sesame, oat, and yam milk (Atalar et al. 2021; Bekiroglu et al. 2022; Ghaderi et al. 2021; Butt et al. 2023; Batista et al. 2019).

*Hibiscus sabdariffa L*. (roselle) is rich in polyphenolic acids, flavonoids, and anthocyanins, giving it high antioxidant capacity (Mohagheghi et al. 2011). It has been incorporated into products such as yogurt, jelly candy, and meat formulations (Arslaner et al. 2021; Halim et al. 2022; Santos et al. 2022).

Probiotics, when consumed in adequate amounts, provide health benefits to the host. However, their viability can be reduced during processing, storage, and digestion. Encapsulation techniques, particularly extrusion, have been shown to enhance probiotic stability while enabling co‐delivery with bioactive compounds (Misra et al. 2021; Agriopoulou et al. 2023). Co‐encapsulation of probiotics with plant‐derived antioxidants, such as onion peel, beetroot, pomegranate peel, or rose hip extract, has improved bacterial survival (Ejaz et al. 2023; Bautista Villarreal et al. 2023; Al‐Moghazy et al. 2022; Didar 2024).

Despite the growing interest in plant‐based ice cream, no study has yet explored the co‐encapsulation of probiotics with *Hibiscus sabdariffa* extract in a vegan ice cream formulation; therefore, this study aimed to co‐encapsulate *Lactobacillus casei* and *Hibiscus sabdariffa* calyces extract via extrusion, incorporate the microcapsules into sesame milk‐based vegan ice cream, and evaluate the physicochemical properties, probiotic viability during storage, and survival under simulated gastrointestinal conditions.

## 2 Material and Methods

Sesame seed and Hibiscus ( *Hibiscus sabdariffa* ) calyces purchased from a local market (Neyshabur, Iran) are authenticated by the Department of Systematic Plant Biology of the Islamic Azad University of Neyshabur. Cream powder, sugar powder and vanilla also were brought from the local market. The chemicals used in this study were purchased from Merck (Germany). *Lactobacillus casei* (PTCC no: 1608) was purchased from Iranian Research Organization for Science and Technology.

### 2.1 Sesame Milk Preparation

For the preparation of sesame milk, first, sesame was blended with distilled water at a ratio equal to 1:3. Thereafter, it was maintained at room temperature for 16 h. Then, rinsing the sesame with water at a ratio equal to 1:2 was performed and then blanched (15 min at 85°C). The next step was draining and mixing the sesame and distilled water (1:5) in a blender (700 rpm, 20 min). Thereafter, the mixture was kept at room temperature for 1 h and was filtered by a filter cloth and the total dry weight of the sesame milk was regulated to 11%–12% (the final protein content of the sesame milk was 1.8%). The resulting milk was pasteurized in suitable containers at 80°C for 15 s and kept in sealed bags at 4°C–5°C for the next steps (Ghaderi et al. 2021).

### 2.2 Preparation of Probiotic Bacteria

The freeze‐dried form of probiotic culture, *Lactobacillus casei, was* purchased from Iranian Research Organization for Science and Technology. It was kept on De Man Rogosa Sharpe (MRS) broth (Difco, Becton & Dickinson, Sparks, MD, USA) in refrigeration until use. In order to obtain fresh cultures, an aliquot of 100 μL was taken from the refrigerated culture and placed in tubes containing 10 mL of MRS medium; they were incubated at 37°C for 24 h for further ANALyses. For the encapsulation, the cells were recovered by centrifugation at 590 × g for 20 min at 25°C and washed twice with 10 mL of 8.5 g/L of a saline solution. The cells were then suspended in 1 mL of the saline solution and used for the respective encapsulation method (Bautista Villarreal et al. 2023). A plate count method in MRS was used to determine the final count of cells in the suspension (Bautista Villarreal et al. 2023).

### 2.3 Preparation of the Hibiscus ( *Hibiscus sabdariffa* ) Calyces Extract

For the preparation of Hibiscus ( *Hibiscus sabdariffa* ) calyces extract, the method of Bautista Villarreal et al. (2023) was followed. Accordingly, a specific amount of the dried material was soaked in sterile distilled water (20 g/L) and kept at ambient temperature (25°C) for 48 h in a sterile‐capped container. Then, filtration by Whatman No. 1 was carried out and the collected extract was kept in a sterile glass container at 4°C until use. Finally, the extract was incorporated into the capsules based on the following approach.

### 2.4 Encapsulation by Extrusion

For encapsulation through extrusion, the method outlined by Ejaz et al. (2023) and Bautista Villarreal et al. (2023) were performed. For this reason, 1 mL (10^12^ CFU/mL) of the harvested probiotic culture was blended with 99 mL of a specific concentration of alginate solution (10 g/L) that was previously prepared and sterilized by autoclave. Before this step, the incorporation of the extract was done (10 mL) of the plant extract was added to the alginate solution (89 mL). The prepared suspension was extruded dropwise with a syringe through needles in sterile 0.15 M CaCl_2_ with gentle magnetic stirring (Bautista Villarreal et al. 2023). The next steps were filtration by Whatman filter paper and washing with distilled water (Ejaz et al. 2023).

### 2.5 Preparation of Sesame Milk Ice Cream

The formulation used for the preparation of control sesame milk ice cream was according to Alkashouty et al. (2023) and includes 450 g sesame milk, 100 g cream powder, 150 g sugar powder and 3 g vanilla (Alkashouty et al. 2023).

The liquid blend, composed of sesame milk, was continuously mixed under moderate heating, up to 45°C–50°C. Thereafter, the solid substances (sugar, cream powder and vanilla), were added to the heated liquid and dissolved. Then, the mixture was stirred (3 min) and pasteurization was performed (80°C for 25 s). The next step was homogenization by a homogenizer (Ultra Turrax T25D IKA, Germany) at 15,000 rpm for 3 min. Then, the mixture instantly cooled to 5°C with a bath of water, ice, and salt. Afterward, the aging step was carried out at 5°C ± 0.5°C for 24 h. The fabricated ice cream mixture was placed in a batch ice cream maker (Model ICK 5000, Delonghi, Germany) during freezing for 20 min. Finally, the samples are placed into plastic containers, coded, and stored at −18°C for at least 24 h (Ghaderi et al. 2021).

The preparation of probiotic ice cream was carried out similarly and co‐encapsulated probiotics were added before freezing in order to achieve approximately 10^8^ CFU/g (Jurkiewicz et al. 2011).

### 2.6 Characterization of Physicochemical Properties of Ice Cream Samples

The amount of fat was being measured by the Gerber method (Kurt and Atalar 2018).

The overrun was assessed following the method outlined by Batista et al. (2019). The mass of the ice cream mixture and the mass of the ice cream were determined and overrun was calculated by the following equation:

$$\text{Overrun}\;\left(\%\right)=\frac{\text{Volume of icecream mixture}-\text{volume of}\;\mathrm{ice}\;\text{cream}}{\text{Volume of}\;\mathrm{ice}\;\text{cream}}\times 100$$

The magnitude of the melting of the ice cream was assessed according to the method of Hassan and Barakat (2018). First allow 25 g of sample to melt at room temperature 22°C ± 1°C. The samples were placed on a narrow wire screen which had been placed over a glass funnel and the dripping was collected in a beaker. The time of first drop was recorded as melting temperature. The weight of drainage was determined at 45 and 90 min. The percentage of the relative melted amount during each period to determine the melting resistance was calculated.

### 2.7 Rheological Function

Rheological analysis was carried out on the ice cream blends applying a rotational viscometer (Model RVDV‐II, Brookfield Engineering Inc., USA) equipped with a heating circulator (Julabo, Model F12‐MC, Julabo Labortechnik, Germany) at 25°C ± 0.1°C. To eliminate the time dependency, before the measurement, the samples were sheared at 150 s^−1^ for 800 s. Thereafter, the flow behavior data were achieved by applying an enhancing shear rate trend (from 1 to 85 s^−1^) and described by the Herschel‐Bulkley model (Equation 1).

(1) $$\tau =\tau_{o}+{\text{𝑘𝛾’}}^{\text{𝑛}}$$

where τ_0_ is the Herschel‐Bulkley (H‐B) yield stress (Pa), stands for the H‐B consistency coefficient (Pa.s^n^) and depicts the H‐B flow behavior index (dimensionless) (Khosrow Shahi et al. 2021).

### 2.8 Anthocyanin and Flavonol Contents

Determination of total flavonol was carried out according to the method of Lees and Francis (1972).

The total anthocyanin content was measured by the pH differential approach outlined by Lee et al. (2005) applying two buffer systems: potassium chloride buffer, pH 1 (0.025 M), and sodium acetate buffer, pH 4.5 (0.4 M). Samples were diluted in pH 1.0 and pH 4.5 buffers and absorbance measurements were made at 510 and 700 nm applying 1 cm path length cuvettes. The pigment content was determined and expressed as cyanidin 3‐ glucoside (Cyd 3‐ glu) per 100 g FW, using an extinction coefficient (e) of 26,900 L cm^−1^ mol^−1^ and a molar weight of 449.2 g mol^−1^

$$\text{Absorbance}\;\left(A\right)=\left(A_{51\text{0nm}}-A_{70\text{0nm}}\right)\;\mathrm{pH}.1.0–\left(A_{51\text{0nm}}-A_{70\text{0nm}}\right)\;\mathrm{pH}.4.5$$

Monomeric anthocyanin contents (mg L^−1^) were measured according to the following equation:

$$\mathrm{ACN}=\frac{A\times \mathrm{MW}\times \mathrm{DF}\times 1000}{e\times 1}$$

where A, absorbance; MW, molar weight (449.2); DF, dilution factor; *e*, molar absorptivity (26,900) (Lee et al. 2005; Shamshad et al. 2023).

### 2.9 Fourier Transform Infrared (FTIR) Spectroscopy

The FTIR spectrum of sodium alginate, Hibiscus ( *Hibiscus sabdariffa* ) calyces extract, and co‐extruded microcapsules containing the extract was performed at a wavelength equal to 4000–400 cm^−1^ by Thermo, AVATAR model, USA.

### 2.10 Evaluating the Texture of the Ice Cream

The hardness magnitude of ice cream as an indicator of the texture property of samples was measured by Texture Analyzer (TA‐XT2 Stable Micro Systems Co. Ltd., Surrey, UK). First, samples were kept at −18°C for 24 h. The condition of the performed test was as follows: A cylindrical probe (5 mm diameter) was attached to a 30 kg load cell and the penetration depth at the geometrical center of the samples was 10 mm; the penetration rate was 1.0 mm/s. The hardness is considered the peak pressure force (g) during penetration (Kurt and Atalar 2018).

### 2.11 Determination of Total Phenolic Compounds and Antioxidant Activity

The total concentration of phenols in the samples was determined according to Gheisari et al. (2020) applying Folin‐Ciocalteu (F‐C) colorimetric approach. Approximately, 5 g of ice cream samples was extracted in 25 mL of methyl alcohol (Merck, Germany). After 12 h refrigeration, the extracts were filtered (Whatman No. 4) and used for the measurement of phenolics and DPPH) 2,2‐diphenyl‐1‐picrylhydrazyl (scavenging activity. Extracts (0.2 mL) were blended with 1.8 mL of distilled water, 1 mL of F‐C reagent (Sigma‐Aldrich, USA), and 2 mL of 20% sodium carbonate (Merck, Germany). The mixture was maintained at 25°C for 20 min, and the absorbance was determined at 735 nm. The various concentrations of gallic acid (GA) (0, 0.1, 0.25, 0.5, 0.75, and 1 mg/mL) were applied for calibration curve, and the results were expressed as mg GAE/100 g of sample (Gheisari et al. 2020).

The extract (0.3 mL) obtained in the previous step was blended with 1.2 mL methanol and 1.5 mL DPPH solution in methanol (0.5 mmol/L). After 90 min of reaction at room temperature, the sample absorbance was determined at 515 nm. Methanol was used as a blank. The DPPH scavenging activity was calculated as shown below (Gheisari et al. 2020):

$$\%\text{DPPH scavenging}=\frac{\text{absorbance control}-\text{absorbance sample}\;}{\text{absorbance control}}\times 100$$

### 2.12 Characterization of Morphology of Microcapsules by Optical Microscopy

For the assessment of the morphology of microcapsules, a Stereo Microscope (Olympus, BX41) was applied with magnification 40× and 10×.

### 2.13 Viability Rate of Probiotic Bacteria in Samples on Simulated Gastrointestinal Condition

For evaluation of the viability rate of probiotic bacteria in simulated gastrointestinal circumstances, 1 g of ice cream sample was homogenized with 9 mL of sterile electrolyte mixture (6.2 g/L NaCl, 2.2 g/L KCl, 1.2 g/L NaHCO_3_, 0.22 g/L CaCl_2_, and lysozyme 0.01%) in a Stomacher blender for 3 min. Thereafter, 10 mL of electrolyte blend containing 0.3% pepsin was mixed into the solution. The pH was reached to 2.0 by HCl solution (1.0 mol/L), and this blend was incubated at 37°C for 1 h. In the simulated intestinal phase, the pH was enhanced to 7.5 with a sterile alkaline solution including bile (10 g/L) and pancreatin (1 g/L). Finally, the mixture was incubated under anaerobic circumstances at 37°C for 4 h (Haghani et al. 2021).

### 2.14 Assessment of the Viability of Probiotic Cells

The number of probiotic cells in the ice cream samples after simulated gastrointestinal and frozen storage time (1, 7, 14, 30, and 45 days) of frozen storage was enumerated as follows: 10 g of each sample was diluted and homogenized in 90 mL of peptone water. Thereafter, serial decimal dilutions were performed. 0.1 mL of the mixture was inoculated on Petri plates of MRS agar by the pour‐plating approach. The plates were incubated anaerobically (48 h, 37°C). The number of cells was expressed as log CFU/g (Haghani et al. 2021; Kowalczyk et al. 2022).

### 2.15 Analysis of Organoleptic Properties of Ice Cream Samples

The ice cream samples were kept at −18°C for 30 d, and organoleptic evaluation was done by the contribution of 10 trained panelists (a team that included common consumers of sesame seed and dairy products) by the hedonic method. The trained panelists undergo rigorous training to recognize and accurately assess specific sensory attributes. Training involves enhancing the panelists' proficiency in accurately identifying and describing attributes with precision, following standardized procedures and criteria (Bhuker and Maurya 2024). The assessed properties included appearance/color, texture, smoothness, melting resistance, flavor, creaminess, foreign taste and general acceptability. The score for each property was from 1: undesirable to 10: desirable (Karaman et al. 2014).

### 2.16 Statistical Analysis

All experiments were accomplished three times. Data were analyzed using ANOVA, SPSS version 24.0.0. All data are expressed as mean ± standard deviation. A value of *p* < 0.05 was considered significant.

## 3 Results and Discussion

### 3.1 Characterization of Microcapsules

#### 3.1.1 Microscopic Characterization of Microcapsules

**FIGURE 1:**
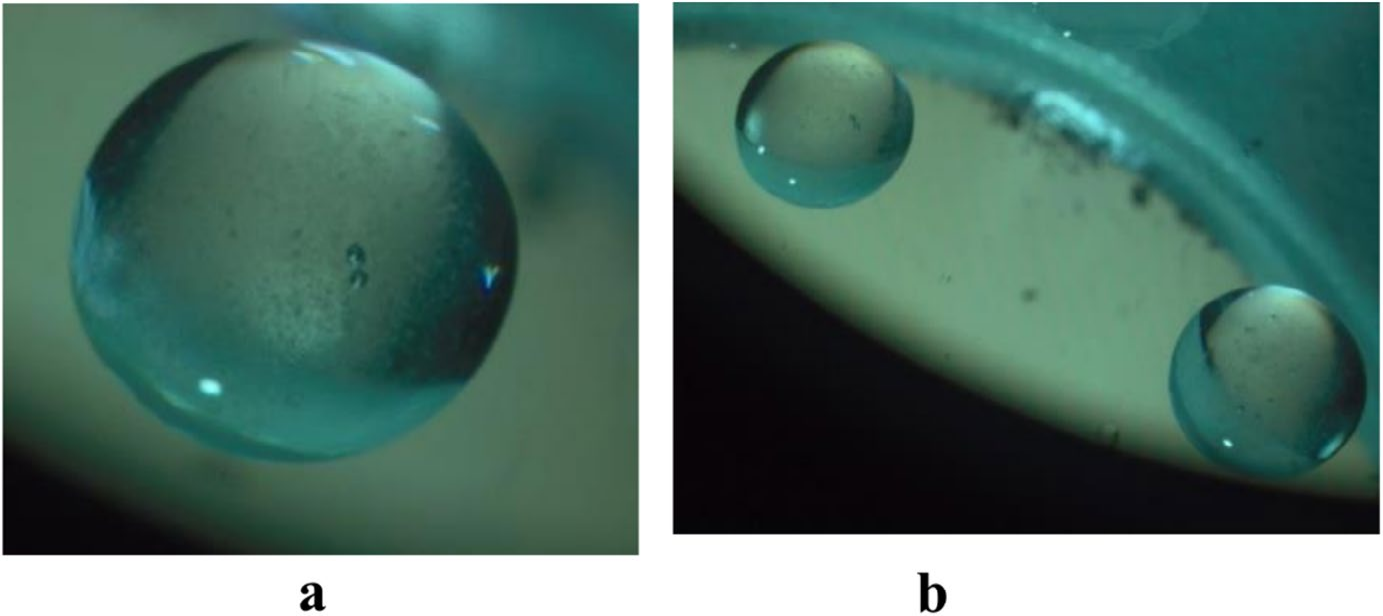
Optical microscopy of microcapsules at magnification 40× (a) and 10× (b).

Observation of the microcapsule structure was performed by the stereo microscope model Olympus, BX41, depicted in Figure 1a,b.

Observation under the stereo microscope (Olympus BX41) showed that the alginate‐based probiotic microcapsules were predominantly spherical with a mean diameter of 502 ± 18 μm (*n* = 50), consistent with the droplet extrusion parameters used. No visible cracks or pores were detected at the magnifications employed, suggesting structural integrity and potential for good encapsulation efficiency. The mean size obtained here is comparable to that reported by Ejaz et al. (2023), who produced alginate beads of 480–520 μm using a similar extrusion setup (Ejaz et al. 2023). Bautista Villarreal et al. (2023) also achieved droplets of ~500 μm (Bautista Villarreal et al. 2023). In contrast, Zanjani et al. (2014) reported smaller capsules (350–400 μm) when using a finer gauge needle and lower viscosity alginate solution (Zanjani et al. 2014).

#### 3.1.2 Fourier Transform Infrared Spectroscopy (FTIR)

**FIGURE 2:**
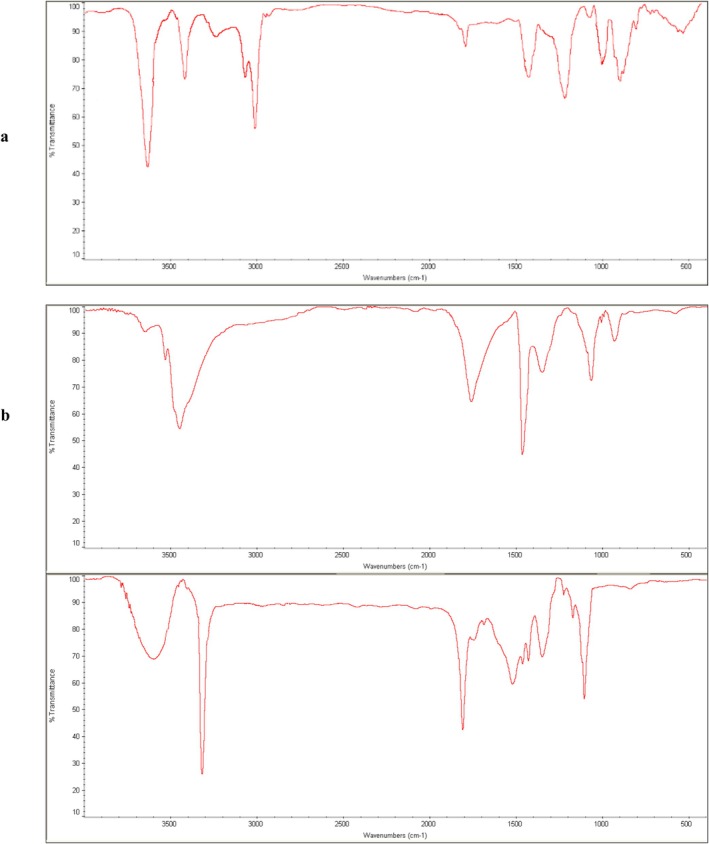
Fourier Transform Infrared (FTIR) spectra of: (a) sodium alginate, (b) Hibiscus ( *Hibiscus sabdariffa* ) calyces extract, and (c) co‐extruded microcapsules containing the extract. Spectra were recorded in the range 4000–400 cm^−1^.

FTIR analysis of alginate, Hibiscus ( *Hibiscus sabdariffa* ) calyces extract, and microcapsules was performed and the results are shown in Figure 2a–c. Accordingly, the basic peaks of alginate appeared at 3450, 1760, 1420 and 1068 cm^−1^ which could be ascending to the presence of OH, C=O, OH and C‐O bands, respectively (*Libretex Chemistry* n.d.). The presence of OH, COO and CO groups in FTIR of sodium alginate also was reported by Pereira et al. (2011).

Considering the FTIR spectra of Hibiscus ( *Hibiscus sabdariffa* ) calyces extract, it is obvious that the major peaks appeared at 3587, 3420, and 1730 cm^−1^ which could be attributed to the O‐H stretch vibrations (phenols), H‐bonded (flavonoids), O‐H stretch (carboxylic acids), and C=O stretch. Other remarkable peaks appeared at 1364 and 928 cm^−1^ that might be due to C‐H (alkanes), and =C‐H bond (alkenes) (Ityo et al. 2023). These functional groups indicate the presence of antioxidant phytochemicals such as polyphenols and organic acids, which can contribute to oxidative stability in food systems and potentially interact with alginate chains through hydrogen bonding. In the case of co‐extruded microcapsules, the major peaks appeared at 3600, 3300, 1750, and 1419 cm^−1^ which could be ascending to the O‐H stretch vibrations (alcohols), H‐bonded (flavonoids), C=O stretch (Ityo et al. 2023), and symmetric stretching vibration of COO groups (Pereira et al. 2011). Other remarkable peaks were observed at 1370, 1300, and 1050 cm^−1^ which could be attributed to the C‐H (alkanes), C‐O stretch (Ityo et al. 2023) and elongation of C‐O groups, respectively (Pereira et al. 2011). The retention of characteristic peaks from both alginate and *Hibiscus* extract in the microcapsules confirms the successful incorporation of the bioactive compounds into the alginate matrix without significant chemical degradation.

### 3.2 Measurement of Physicochemical Properties of Sesame Milk Ice Cream

Physicochemical characterization of prepared ice cream samples was performed, and the outcomes are shown in Table 1.

**TABLE 1:** Physicochemical properties of sesame milk ice cream.

Sample	Fat (%)	Hardness (*N*)	Overrun (%)	Melting resistance (%)	Anthocyanin content (mg/100 g)	Total flavonol content (mg/100 g)	Ascorbic acid content (mg/100 g)	TPC (mg GAE/100 g)	Antioxidant activity (%)
Control	2.1 ± 0.1^a^	98 ± 2^a^	22.42 ± 1.2^b^	80.2 ± 0.5^b^	ND	0.7 ± 0.01^b^	0.06 ± 0.01^b^	0.25 ± 0.02 ^b^	10.5 ± 2.5^b^
Enriched sample	2.1 ± 0.2^a^	90 ± 2^b^	28.42 ± 1.5^a^	88.2 ± 0.2^a^	0.25 ± 0.01^a^	7.57 ± 0.1^a^	0.14 ± 0.01^a^	3.72 ± 0.3 ^a^	31 ± 1.2^a^

*Note:* Different superscript lowercase letters show significant differences between the samples (*p* < 0.05).

Abbreviation: ND, not detectable.

The fat content of both control and enriched ice cream samples was approximately 2.1%, with no significant difference between them (*p* > 0.05). This value is higher than the 1.01% ± 0.01% reported by Nateghi et al. (2018) for ice cream formulated with 60 g sesame milk and 35 g hemp seed milk (Nateghi et al. 2018), likely due to the different raw material ratios and formulations used in the present study.

Hardness values were 98 ± 2 N for the control and 90 ± 2 N for the enriched sample, indicating a significant reduction in hardness upon enrichment (*p* < 0.05). This decrease coincided with a higher overrun in the enriched ice cream, supporting the inverse relationship between overrun and hardness previously observed by Liu et al. (2023), who found that high‐overrun ice creams (~90%) had the lowest hardness, whereas reduced overrun (~30%) increased firmness (Liu et al. 2023). In our case, the enriched sample's higher overrun contributed directly to its softer texture.

The melting resistance of the enriched ice cream (88.2% ± 0.2%) was higher than that of the control (80.2% ± 0.5%), indicating a significant increase in resistance to melting (*p* < 0.05). This enhancement may be related to the higher anthocyanin and polyphenol contents in the enriched formulation, which can improve structural stability. Dara et al. (2024) similarly observed that incorporating pigmented barberry anthocyanins reduced melting rates in ice cream by increasing air entrapment and altering protein–polyphenol interactions. These interactions promote the formation of a stable gel matrix capable of holding air bubbles, fat globules, and ice crystals, thereby maintaining shape even as ice begins to melt (Dara et al. 2024). Our results support this mechanism, suggesting that the polyphenolic components introduced via enrichment contributed to the observed increase in melting resistance.

The overrun of the enriched sample (28.42% ± 1.5%) was significantly higher than that of the control (22.42% ± 1.2%) (*p* < 0.05), indicating that enrichment increased the incorporation of air during freezing (Table 1). This increase likely contributed to the improved melting resistance observed in the enriched sample. Similar trends were reported by Dara et al. (2024), who found that higher levels of copigmented barberry anthocyanins expanded ice cream volume and increased air content (Dara et al. 2024). The insulating effect of this trapped air can slow melting, as also noted by Warren and Hartel (2018), who observed that high‐overrun ice creams demonstrated better melting resistance due to the thermal insulation provided by air cells (Warren and Hartel 2018).

The enriched ice cream contained 0.25 ± 0.01 mg anthocyanin/100 g, confirming successful incorporation of anthocyanin‐rich ingredients. The presence of anthocyanins in *H. sabdariffa* is well established, with content influenced by extraction conditions (Maciel et al. 2018) and method, where integrated ScCO_2_ and SWE yielded higher anthocyanin recovery (Rizkiyah et al. 2023).

Total flavonol content increased markedly from 0.7 ± 0.01 mg/100 g in the control to 7.57 ± 0.1 mg/100 g in the enriched sample. Similar compounds have been reported in sesame seeds, with white varieties containing 4.34 ± 0.1 to 4.99 ± 0.03 mg/g total flavonoid content (Agidew et al. 2021), and in *H. sabdariffa* calyces, where levels range from 2.50 ± 0.08 to 4.03 ± 0.14 μg/mg depending on source and solvent (Rizki et al. 2017).

Ascorbic acid content was also higher in the enriched ice cream (0.14 ± 0.01 mg/100 g) than in the control (0.06 ± 0.01 mg/100 g). Comparable values have been found in sesame seeds (~0.08 mg/100 g) (Abdiani et al. 2024) and *H. sabdariffa* calyces (Peter et al. 2014). These increases in bioactive compounds in the enriched sample are consistent with the inclusion of Hibiscus calyces and sesame derivatives, both known sources of anthocyanins, flavonoids, and vitamin C.

The enriched ice cream had a total phenolic content of 3.72 ± 0.3 mg GAE/100 g, markedly higher than the control (0.25 ± 0.02 mg GAE/100 g), confirming the phenolic contribution of the added Hibiscus–sesame components. Sharma et al. (2016) reported presence of phenolic compounds in sesame seed (Sharma et al. 2016). The presence of phenolic compounds such as chlorogenic, protocatechuic, caffeic, and gallic acids previously identified in *Hibiscus sabdariffa* calyces (Ayubi et al. 2024).

Antioxidant activity also increased significantly from 10.5% ± 2.5% in the control to 31% ± 1.2% in the enriched sample (*p* < 0.05). This enhancement is consistent with literature reporting antioxidant activity in sesame seeds (21.49%–32.39%) (Dravie et al. 2020). Roselle calyx contains a rich source of dietary fiber, vitamins, minerals, and bioactive components such as organic acids, phytosterols, and polyphenols, some of which have antioxidant characteristics (Shruthi and Ramachandra 2019). The results indicate that enrichment with these plant‐derived components effectively boosts both phenolic content and antioxidant potential in the final product.

### 3.3 Viscosity

**FIGURE 3:**
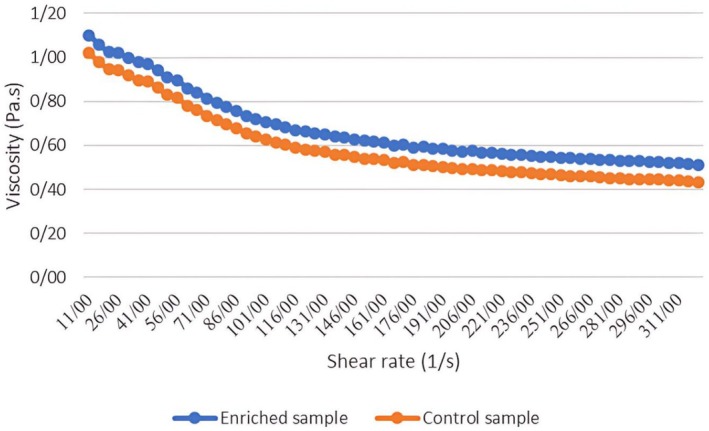
Flow behavior diagram of two samples of ice cream (control and enriched).

The flow behavior curves (Figure 3) show that viscosity decreased with increasing shear rate for both the control and enriched ice cream samples. A similar trend was reported by Hou (2017) for sesame paste with various added water (Hou 2017). The enriched formulation consistently exhibited higher viscosity than the control. The viscosity in ice cream blends has an impact on overrun, creaminess, and melt resistance by improving air retention and slowing heat transfer (Shadordizadeh et al. 2023). Our findings are consistent with the observations of Shadordizadeh et al. (2023), who reported that increasing the content of encapsulated *Indigofera tinctoria* extract elevated the consistency coefficient (k) due to greater dry matter content (Shadordizadeh et al. 2023). These parallels suggest that the higher viscosity in our enriched sample is likely related to its increased solids and bioactive content, which may promote more extensive structural networking within the mix.

### 3.4 Survival of Probiotic Bacteria in Ice Cream Samples Under Simulated Gastrointestinal Condition

The simulated gastric juice test (pH 2) revealed that the logarithmic number of viable counts of co‐extruded *L. casei* decreased from 8 to 7.1 log CFU/g after 1 h, indicating that although acid exposure reduced viability, a substantial proportion of the probiotics survived. This finding highlights the protective role of co‐extrusion under harsh gastric conditions (Afzaal et al. 2022). Comparable reductions have been reported in yogurt ice cream containing *Bifidobacterium longum subsp. longum* TISTR 2195 during in vitro gastric simulation, where low pH was identified as the primary stress factor (Toommuangpak and Thaiudom 2024). The enhanced survival observed in our study aligns with Afzaal et al. (2022), who found that encapsulation improved probiotic tolerance to gastric acidity compared to free cells (Afzaal et al. 2022).

In simulated intestinal juice (pH 7.5), the logarithmic number of viable count further decreased to 6.2 log CFU/g. Although viability declined, the reduction was smaller than that reported for free probiotic cells in similar conditions (Afzaal et al. 2020). Toommuangpak and Thaiudom (2024) also observed reduced probiotic counts in intestinal conditions due to bile salt–induced damage to cell walls, particularly in Gram‐positive bacteria (Toommuangpak and Thaiudom 2024). The higher final count in our enriched ice cream compared with these studies may indicate that encapsulation mitigated bile salt stress, supporting previous findings on calcium alginate and whey protein concentrate encapsulation (Afzaal et al. 2022).

Additionally, the presence of bioactive‐rich ingredients such as *Hibiscus sabdariffa* calyces may have contributed to probiotic protection. Haghani et al. (2021) reported that bioactives, fibers, and vitamins in cornelian cherry (*Cornus mas L*.) peel enhanced probiotic survival in ice cream (Haghani et al. 2021), and similar phenolic and micronutrient profiles have been documented in *Hibiscus sabdariffa* calyces (Ayubi et al. 2024; Rizki et al. 2017).

### 3.5 Survival of Probiotic Bacteria, Retention of Anthocyanin, TPC, Total Flavonol, and Ascorbic Acid During Storage

The stability of *Lactobacillus casei* , anthocyanins, total phenolics, total flavonols, and ascorbic acid was monitored during 45 days of frozen storage (Table 2).

**TABLE 2:** Evaluation of the stability of probiotic bacteria and various components in enriched ice cream samples during storage.

Storage time (day)	1	7	14	30	45
Probiotic viability (log CFU/g)	8 ± 0.01^a^	8 ± 0.01^a^	7 ± 0.01^b^	6 ± 0.01^c^	6 ± 0.01^c^
Anthocyanin content (mg/100 g)	0.25 ± 0.01^a^	0.22 ± 0.01^b^	0.20 ± 0.01^c^	0.17 ± 0.01^d^	0.14 ± 0.01^e^
Total phenolic content (mg GAE/100 g)	3.72 ± 0.3^a^	3.72 ± 0.3^a^	3.72 ± 0.29^a^	3.72 ± 0.29^a^	3.72 ± 0.28^a^
Total flavonol (mg/100 g)	7.57 ± 0.1^a^	7.56 ± 0.1^a^	7.55 ± 0.1^a^	7.53 ± 0.1^a^	7.51 ± 0.1^a^
Ascorbic acid (mg/100 g)	0.14 ± 0.01^a^	0.1 ± 0.01^b^	0.098 ± 0.01^c^	0.098 ± 0.01^c^	0.098 ± 0.01^c^

*Note:* Different superscript lowercase letters show significant differences between the samples (*p* < 0.05).

#### 3.5.1 Probiotic Viability

The initial viable count (8.00 ± 0.01 log CFU/g) declined to 6.00 ± 0.01 log CFU/g by day 45. This reduction indicates that while some cell loss occurred, counts remained above the recommended therapeutic minimum for probiotics (According to FAO/WHO (2002) recommendation, to exert their positive effect on the host, they must be ingested in adequate amounts, that is, probiotic bacteria should be 10^6^–10^9^ CFU/g in the product at the time of ingestion) (Zielińska et al. 2018). The decline is likely linked to oxygen exposure during freezing, mechanical stress, and ice crystal formation, which can damage cell membranes and cause thermal shock (Haghani et al. 2021; de Souza et al. 2019). Similar viability loss has been observed in *Lactobacillus acidophilus* and *Bifidobacterium lactis* during ice cream storage, with survival further improved by encapsulation with mango peel extract (Hayayumi‐Valdivia et al. 2021).

#### 3.5.2 Anthocyanin Content

Anthocyanin concentration decreased progressively from 0.25 ± 0.01 to 0.14 ± 0.01 mg/100 g over storage. This loss is consistent with reports of anthocyanin degradation in frozen dairy systems, attributed to oxygen exposure, pH sensitivity, and structural breakdown (Shamshad et al. 2023; Boyanova et al. 2022). Anthocyanin decay has also been documented in fruit pulps during frozen storage, with the rate influenced by freezing speed (Borges et al. 2023). The stability in our samples was higher than in some berry‐based (lingonberry extract of 5%) ice creams where total anthocyanins became undetectable after 30 days (Boyanova et al. 2022).

#### 3.5.3 Total Phenolic Content (TPC)

TPC remained stable (3.72 ± 0.3 mg GAE/100 g) throughout storage, indicating strong phenolic stability in ice‐cream samples. This matches Haghani et al. (2021), who found no TPC loss in ice cream enriched with *Cornus mas* peel (Haghani et al. 2021).

#### 3.5.4 Total Flavonols

Flavonol levels showed no significant change (*p* > 0.05), dropping only slightly from 7.57 to 7.51 mg/100 g. This stability is consistent with reports that freezing has minimal effect on flavonoid content in plant‐based foods (Rababah et al. 2023; Mullen et al. 2002).

#### 3.5.5 Ascorbic Acid

Vitamin C declined from 0.14 ± 0.01 to 0.098 ± 0.01 mg/100 g, with the largest decrease occurring within the first 14 days. This pattern is consistent with observations in frozen strawberries (Sahari et al. 2004) where initial rapid losses were attributed the increase in concentration of solutes that happened in the unfrozen phase during freezing (Sahari et al. 2004).

### 3.6 Sensory Evaluation

**FIGURE 4:**
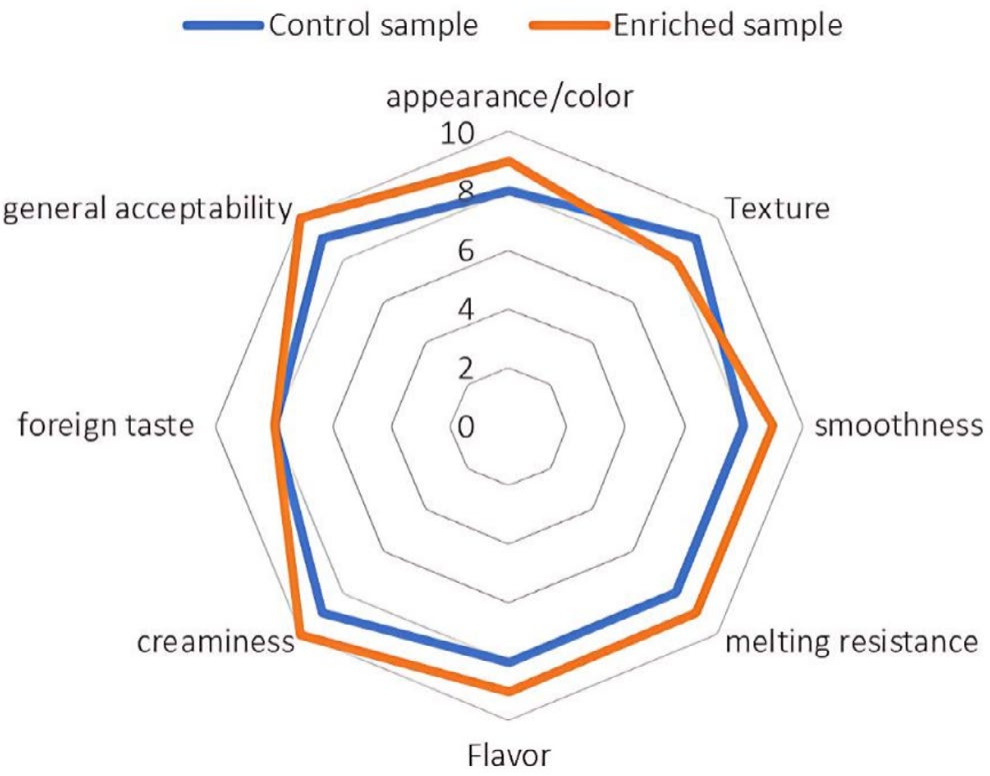
Sensory characterization of control and enriched ice cream samples.

The sensory assessment (10‐point hedonic scale) revealed that enriched ice cream samples demonstrated improved characteristics compared to the control in several attributes (Figure 4).

The enriched samples exhibited slower melting, consistent with their higher melting resistance (88.2% ± 0.2%) compared to the control (80.2% ± 0.5%) (Table 1). Dara et al. (2024) reported similar results in melting resistance in ice creams containing varying percentages of copigmented barberry anthocyanin (Dara et al. 2024).

Texture analysis by the panelists indicated higher hardness in the control sample, consistent with instrumental hardness measurements (Table 1). This suggests that the enrichment process may have slightly softened the ice cream structure. Color and appearance scores were significantly higher in the enriched samples, aligning with their enhanced visual appeal due to the natural pigments in the Hibiscus ( *Hibiscus sabdariffa* ) calyces extract. Likewise, flavor scores improved in the enriched formulation. Dara et al. (2024) also observed increased sensory scores for color and flavor in anthocyanin‐enriched ice creams compared to controls (Dara et al. 2024). Overall acceptability was higher for enriched samples, showing that panelists preferred the enriched formulation over the control (Figure 4).

## 4 Conclusion

The addition of co‐extruded probiotic bacteria ( *Lactobacillus casei* ) and Hibiscus ( *Hibiscus sabdariffa* ) calyces extract to sesame milk ice cream was evaluated. Accordingly, the addition of the microcapsule increased anthocyanin, flavonol, ascorbic acid, TPC and antioxidant activity in the enriched sample. At the end of the storage time (45 days), the logarithmic count of viable cells reaches 6 6 ± 0.01 CFU/g. The TPC content does not change but other components anthocyanin, total flavonol, and ascorbic acid have significant decreases at the end of the storage time (*p* < 0.05). At the simulated gastrointestinal conditions, the viable log count of co‐extruded probiotics bacterial cells decreased and at the end of this stage, the logarithmic number of probiotic cells reached 6.2. Sensory characterization of both ice cream samples showed, there were significant differences between several sensory properties of control and enriched samples (*p* < 0.05) and the enriched samples had higher scores in appearance, flavor, and general acceptability. The results of this study demonstrate that the formulation successfully maintained probiotic viability above the recommended minimum threshold for functional foods (≥ 6 log CFU/g) after 45 days of frozen storage, while delivering measurable levels of anthocyanins, flavonols, ascorbic acid, and phenolic compounds with antioxidant potential. However, the study was limited by the use of a single probiotic strain, one plant extract source and a relatively short storage duration (45 days).

## Author Contributions

Conceptualization: Zohreh Didar. Data curation: Zohreh Didar and Mohammad Hossein Haddad Khodaparast. Formal analysis: Zohreh Didar and Mohammad Hossein Haddad Khodaparast. Funding acquisition: Zohreh Didar. Investigation: Zohreh Didar and Mohammad Hossein Haddad Khodaparast. Methodology: Zohreh Didar and Mohammad Hossein Haddad Khodaparast. Project administration: Zohreh Didar and Mohammad Hossein Haddad Khodaparast. Resources: Zohreh Didar and Mohammad Hossein Haddad Khodaparast. Software: Zohreh Didar and Mohammad Hossein Haddad Khodaparast. Supervision: Zohreh Didar. Validation: Zohreh Didar. Visualization: Zohreh Didar. Writing – original draft (equal); writing – review and editing: Zohreh Didar and Mohammad Hossein Haddad Khodaparast, and Behzad Goharjoo.

## Funding

The authors have nothing to report.

## Ethics Statement

The authors have nothing to report.

## Consent

The authors have read and agreed to the published version of the manuscript.

## Conflicts of Interest

The authors declare no conflicts of interest.

## Data Availability

The data that support the findings of this study are available on request from the corresponding author.
